# Supplementary material for: Timing of ICSI with Respect to Meiotic Spindle Status
Source: Int J Mol Sci. 2022 Dec 21;24(1):105. doi: 10.3390/ijms24010105 (PMC9820079; doi:10.3390/ijms24010105)
Supplement: Supplementary file 1 [file ijms-24-00105-s001.zip › ijms-2084387-supplementary.pdf]

International Journal of *Molecular Sciences*

MDPI

Supplementary Material

Timing of ICSI with respect to meiotic spindle status

Olga Tepla [1], Zinovij Topurko [1], Simona Jirsova [1], Martina Moosova [1], Eva Fajmonova [1], Radek Cabela [1], Katerina Komrskova [2,3,*], Irena Kratochvilova [4,*] and Jaromir Masata [1]

[1] Department of Obstetrics and Gynecology of the First Faculty of Medicine and General Teaching Hospital, Apolinarska 18, 128 51 Prague 2, Czech Republic

[2] Laboratory of Reproductive Biology, Institute of Biotechnology of the Czech Academy of Sciences, BIOCEV, Prumyslova 595, 252 50 Vestec, Czech Republic

[3] Department of Zoology, Faculty of Science, Charles University, Vinicna 7, 128 44 Prague 2, Czech Republic

[4] Institute of Physics of the Czech Academy of Sciences, Na Slovance 2, CZ-182 21, Prague 8, Czech Republic

*Correspondence: JM: jaromir.masata@vfn.cz; KK: katerina.komrskova@ibt.cas.cz; IK: krat@fzu.cz

Citation: To be added by editorial staff during production.

Academic Editor: Firstname Lastname

Received: date

Accepted: date

Published: date

Publisher's Note: MDPI stays neutral with regard to jurisdictional claims in published maps and institutional affiliations.

Copyright: © 2022 by the authors. Submitted for possible open access publication under the terms and conditions of the Creative Commons Attribution (CC BY) license (https://creativecommons.org/licenses/by/4.0/).

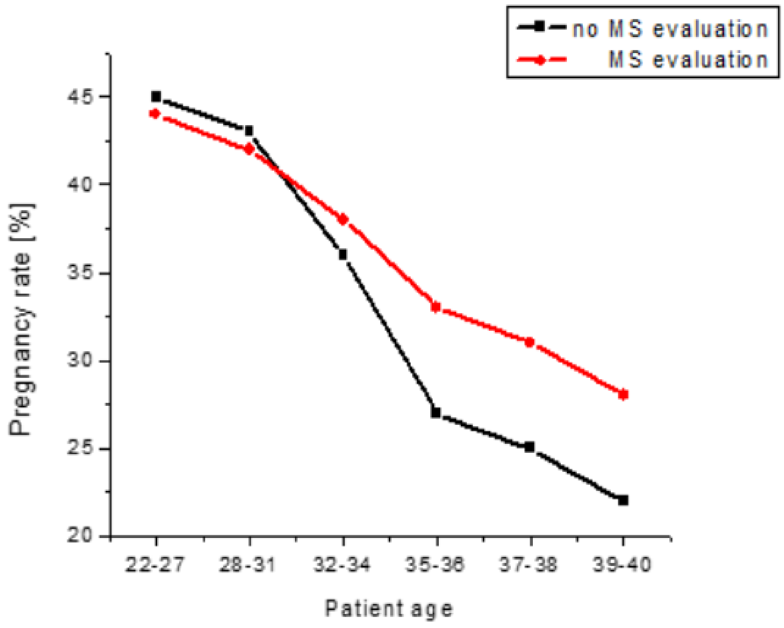

Figure S1. Dependence of patient's pregnancy rate on age for patients treated with MS evaluation and without MS evaluation. The patients were divided into two age dependent groups: 1. Group of patients over 35 years ((≥ 35 years); and 2. Group of patients younger than 35 years (< 35 years). The reason was an important change in the pregnancy rate around the patient's age of 35. Also, for patients older than 35 years MS evaluation began to play an important role in pregnancy rate. In all cases, the pregnancy rate results were calculated for the closest as possible number of patients in compared groups, the differences between pregnancy rates were significant at $p < 0.1$.

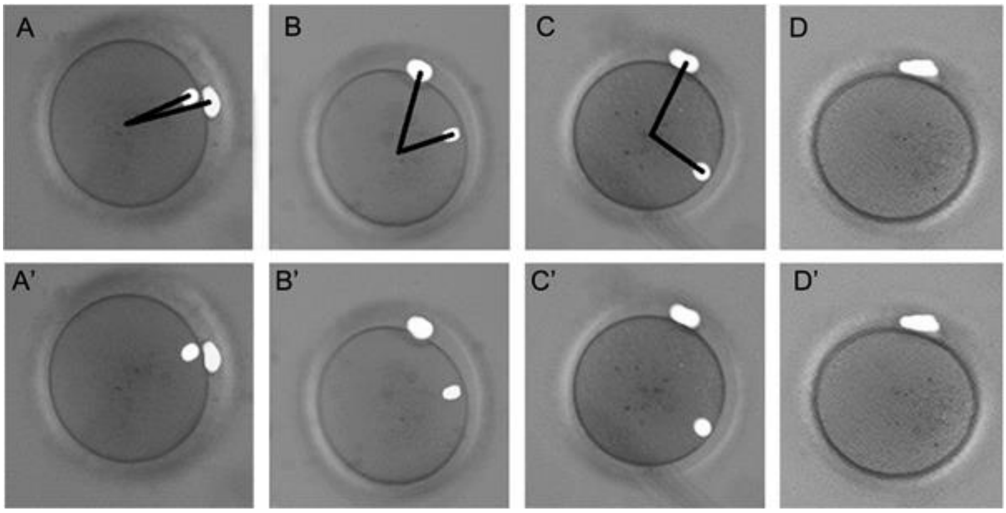

Figure S2. Visualization of MS in polarized light and angles α between MS and PB. Top (A-D): the birefringent MS obtained by polarization microscopy with white highlighted PB and MS. Bottom (A′-D′): the birefringent MS obtained by polarization microscopy with α angles between MS and PB illustrated in black. A: $\alpha < 5°$, B: $\alpha \approx 45°$, C: $\alpha > 60°$, D: invisible MS. Microscopic images were taken at ×100 magnification.

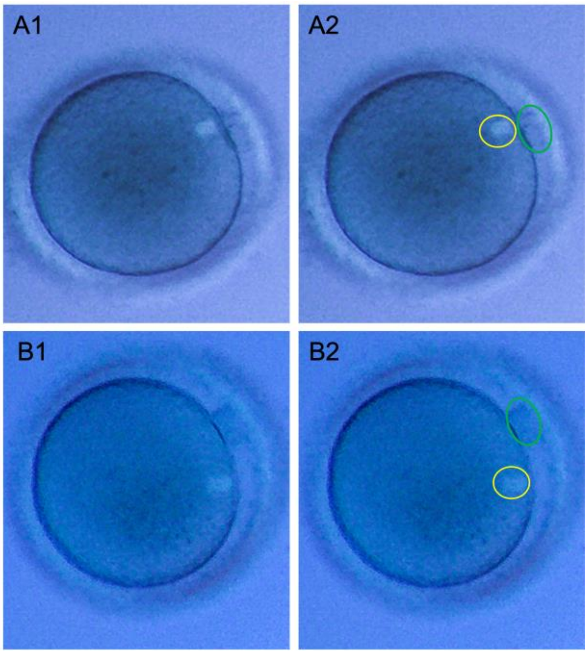

Figure S3. Oocyte maturation indicated by proximity of MS and PB, captured by polarized light microscopy and presented without postprocessing adjustments. A1: the oocyte with MS and PB in close proximity, raw data, A2: highlighted MS (yellow oval) and PB (green oval), B1: the same oocyte after two hours maturation showing the increased α between MS and PB, raw data B2: highlighted MS (yellow). Microscopic images were taken at ×100 magnification.

The average oocyte utilization rate of group of patients younger than 40 years with MS visualization was 53% in comparison to 45% for patients younger than 40 years treated without MS visualization. The difference between in utilization rates was significant at $p < 0.1$ (The value of z was 2.0824. The value of p was .03752).

37 % of patients ≥ 35 years treated with MS evaluation with ICSI performed typically up to 2-3 hours after MS evaluation (5-6 hours after OPU) became pregnant, their average utilization rate was 52% (32/86). 27% patients > 35 years with ICSI typically up to 4-5 hours after MS evaluation (8-9 hours after OPU) became pregnant (26/96 patients), their average utilization rate was 56%. The differences in pregnancy rates between these subgroups of patients older than 35 years was significant at $p < 0.1$ (The value of z was 1.4. The value of p was 0.07). Patients younger than 35 whose oocytes were evaluated by MS visualization and fertilized (ICSI) typically 4-5 hours post MS evaluation (8-9 hours after OPU) had a pregnancy rate of 43 % (29/67). Their average utilization rate was 50%. Patients younger than 35 whose oocytes were evaluated by MS visualization and fertilized up to 2-3 hours post MS evaluation, 5-6 hours after OPU had a pregnancy rate of 40% (29/73), and their average utilization rate was 48%. The differences in pregnancy rates between these subgroups of patients was not statistically significant (The value of z was 0.36. The value of p was 0.36).

The average age of patients evaluated by MS visualization and fertilized (ICSI) typically 8-9 hours after OPU was 35.9 years, while the average age of patients evaluated by MS visualization and fertilized (ICSI) typically 5-6 hours after OPU was 34.8 years. 56% of patients evaluated by MS visualization were fertilized (ICSI) typically 5-6 hours after OPU.

Table S1. Correlation matrixes for selected data sets of patients ≥ 35 years with calculated correlation coefficients.

	**ICSI timing**	**Sperm quality**	**Utilization rate**	**Pregnancy rate**	**GV oocytes**	**M1 oocytes**
**ICSI timing**	1	-0.03 ($p>0.05$)	0.3 ($p>0.05$)	0.35 ($p<0.05$)	0.032 ($p>0.05$)	-0.02 ($p>0.05$)
**Sperm quality**	-0.03 ($p>0.05$)	1	0.02 ($p>0.05$)	0.03 ($p>0.05$)	0.01($p>0.05$)	0.03 ($p>0.05$)
**Utilization rate**	0.3 ($p>0.05$)	0.02 ($p>0.05$)	1	0.4 ($p<0.05$)	0.04 ($p>0.05$)	0.2 ($p>0.05$)
**Pregnancy rate**	0.35 ($p< 0.05$)	-0.03 ($p>0.05$)	0.4 ($p<0.05$)	1	0.1($p>0.05$)	0.15 ($p>0.05$)
**GV oocytes**	0.03 ($p>0.05$)	0.01($p>0.05$)	0.04 ($p>0.05$)	0.1 ($p>0.05$)	1	0.3 ($p>0.05$)
**M1 oocytes**	-0.02 ($p>0.05$)	0.03 ($p>0.05$)	0.2 ($p>0.05$)	0.15 ($p>0.05$)	0.3 ($p>0.05$)	1

Table S2. Correlation matrixes for selected data sets of patients < 35 years with calculated correlation coefficients.

	**ICSI timing**	**Sperm quality**	**Utilization rate**	**Pregnancy rate**	**GV oocytes**	**M1 oocytes**
**ICSI timing**	1	-0.02 (p>0.05)	0.14 (p>0.05)	0.18 (p< 0.05)	0.05 (p>0.05)	-0.05 (p>0.05)
**Sperm quality**	-0.02 (p>0.05)	1	0.02 (p>0.05)	-0.019 (p>0.05)	0.03 (p>0.05)	0.02 (p>0.05)
**Utilization rate**	0.14 (p>0.05)	0.02 (p>0.05)	1	0.47 (p<0.05)	0.03 (p>0.05)	0.01 (p>0.05)
**Pregnancy rate**	0.18 (p< 0.05)	-0.02 (p>0.05)	0.47 (p<0.05)	1	0.15 (p>0.05)	0.05 (p>0.05)
**GV oocytes**	-0.05 (p>0.05)	-0.03 (p>0.05)	0.03 (p>0.05)	0.15 (p>0.05)	1	-0.26 (p>0.05)
**M1 oocytes**	-0.05 (p>0.05)	-0.02 (p>0.05)	0.03 (p>0.05)	0.05 (p>0.05)	-0.26 (p>0.05)	1
